# Evaluating methods for combining rare variant data in pathway-based tests of genetic association

**DOI:** 10.1186/1753-6561-5-S9-S48

**Published:** 2011-11-29

**Authors:** Ashley Petersen, Alexandra Sitarik, Alexander Luedtke, Scott Powers, Airat Bekmetjev, Nathan L Tintle

**Affiliations:** 1 Departments of Mathematics, Computer Science, and Statistics, St. Olaf College, 1520 St. Olaf Avenue, Northfield, MN 55057, USA; 2 Department of Mathematics, Wittenberg University, 200 West Ward Street, Springfield, OH 45501, USA; 3 Division of Applied Mathematics, Brown University, 151 Thayer Street, Providence, RI 02912, USA; 4 Department of Statistics and Operations Research, University of North Carolina, 318 Hanes Hall, CB 3260, Chapel Hill, NC 27599-3260, USA; 5 Department of Mathematics, Statistics and Computer Science, Dordt College, 498 4th Ave. NE, Sioux Center, IA 51250, USA

## Abstract

Analyzing sets of genes in genome-wide association studies is a relatively new approach that aims to capitalize on biological knowledge about the interactions of genes in biological pathways. This approach, called pathway analysis or gene set analysis, has not yet been applied to the analysis of rare variants. Applying pathway analysis to rare variants offers two competing approaches. In the first approach rare variant statistics are used to generate *p*-values for each gene (e.g., combined multivariate collapsing [CMC] or weighted-sum [WS]) and the gene-level *p*-values are combined using standard pathway analysis methods (e.g., gene set enrichment analysis or Fisher’s combined probability method). In the second approach, rare variant methods (e.g., CMC and WS) are applied directly to sets of single-nucleotide polymorphisms (SNPs) representing all SNPs within genes in a pathway. In this paper we use simulated phenotype and real next-generation sequencing data from Genetic Analysis Workshop 17 to analyze sets of rare variants using these two competing approaches. The initial results suggest substantial differences in the methods, with Fisher’s combined probability method and the direct application of the WS method yielding the best power. Evidence suggests that the WS method works well in most situations, although Fisher’s method was more likely to be optimal when the number of causal SNPs in the set was low but the risk of the causal SNPs was high.

## Background

Analysis of single-nucleotide polymorphism (SNP) microarray data in genome-wide association studies has traditionally been agnostic because prior biological knowledge about the genome has not been taken into account. However, as the biological knowledge base increases, it is increasingly common to use a priori biological knowledge in the analysis of SNP data. A recently proposed approach to integrate biological knowledge in the analysis of SNP data in genome-wide association studies is pathway or gene set analysis [1-5].Â In pathway analysis, SNPs are associated with genes, and genes are placed into sets (commonly representing biological pathways). Each set is then tested for association with the phenotype. The driving force behind the development of these methods is to increase the power to identify causal SNPs while decreasing the multiple testing penalties that arise when the hundreds of thousands of SNPs typically involved in traditional genome-wide association studies are tested. Increased power is obtained by leveraging knowledge about relationships between SNPs, genes, and sets or pathways. The evaluation of significance at the gene set level allows investigators to identify genotype-phenotype associations that, although not discernible on a SNP-by-SNP basis, are evident through the amalgamation of SNPs into sets.

Pathway analysis methods have been successful in a variety of applications (e.g., expression data, SNP microarray data). However, this approach has yet to be applied to rare variant analysis of next-generation sequence data. A variety of methods have been proposed for the analysis of rare variants [6-9]. All methods use an approach in which genotype information at individual rare variants is combined across variants within a gene to yield a gene-level statistic. Madsen and Browning [6] suggested the possibility of combining rare variant information across a set (pathway) of genes, and this method has been recently applied to common variants [10], but the approach has not yet been implemented in practice on rare variant data. In addition, this approach has not been compared to traditional methods of pathway analysis, which combine information at the gene level into a gene statistic before combining over the pathway.

In this paper we implement pathway analysis using two opposing strategies. In the first strategy we create sets of SNPs by combining all SNPs within genes in a set or pathway of interest, and then we apply recently proposed rare variant methods to these sets. In the second strategy we use rare variant methods to generate a statistic for each gene (combining information on all rare variants in the gene) and then apply traditional pathway analysis approaches to the gene-level statistics. We compare the strategies by evaluating the type I error rate and comparing statistical power under a variety of different scenarios. Comparisons are made using simulated phenotype data and real sequence data made available as part of Genetic Analysis Workshop 17 (GAW17).

## Methods

### Data

All analyses presented here are based on data provided by the organizers of GAW17. The data consist of 697 unrelated individuals from the 1000 Genomes Project genotyped at 24,487 autosomal SNPs with minor allele frequencies (MAFs) ranging from 7.17 × 10^−4^ to 0.499. All SNPs are contained in at least one of 3,205 different genes, and so the data can be considered a mini-exome scan. The organizers of GAW17 simulated a dichotomous phenotype for the 697 individuals. The dichotomous disease phenotype is caused by a combination of measured SNPs (160 SNPs in 36 genes) and unmeasured SNPs. Two-hundred separate simulated phenotype replicates (each based on the same disease model) were produced.

### Gene set construction

To evaluate the effectiveness of different approaches to pathway analysis, we constructed 2,000 sets of 25 genes with varying degrees of association with the phenotype. The 2,000 sets fall into four broad categories: (1) Five hundred sets contain some number *C* (*C* = 5, 10, 15, 20, and 25) of genes known to contain SNPs causally related to the phenotype (100 sets for each value of *C*). These sets were created by first randomly choosing *C* genes from the list of 36 causal genes and then randomly choosing (25 − *C*) genes from the list of noncausal genes. (2) Five hundred sets were created by randomly selecting 25 genes from the list of noncausal genes. (3) Five hundred sets were created by randomly selecting 25 genes from a list of noncausal genes that also did not show evidence of spurious association with the phenotype. Spuriously associated genes are defined as noncausal genes for which the number of replicates (out of 200) that yield a *p*-value less than 0.05 for the gene, using both the combined multivariate collapsing (CMC) and weighted-sum (WS) methods (see [11]), is more than 15. (4) The five hundred sets described in category 1 were modified so that the (25 − *C*) noncausal genes in the set were selected from a list of genes that were also not spurious [11].

### Rare variant methods

We used two different rare variant methods in our analyses: the WS method [6] and the CMC method [7]. Details of our implementations of the WS and CMC methods are provided by Luedtke et al. [11]. One thousand phenotype permutations were used to assess the significance of the WS statistic, whereas for the CMC method we used the asymptotic distribution of Hotelling’s *T*^2^ statistic to assess statistical significance.

### Pathway analysis

The traditional approach to pathway analysis is to first generate gene scores and then to aggregate the gene scores across all genes in a set; an alternative approach is to aggregate SNPs into sets directly. To implement the gene score aggregation approaches, we use the *p*-values generated by applying the WS and CMC methods to each of the 3,205 genes under study and then use one of three different approaches: (1) Gene set enrichment analysis (GSEA; weighted Kolmogorov-Smirnov test) [12] was implemented on the distribution of negative log *p*-values; (2) a Kolmogorov-Smirnov (KS) test was applied directly to the negative log *p*-value distribution of genes in the set and not in the set; (3) Fisher’s combined probability test (Fisher’s method) was also used on the *p*-values of the genes in the set of interest. We also applied the WS and CMC methods directly to sets of SNPs. Significance of GSEA and the WS method is found using phenotype permutation, whereas other methods use asymptotic distributions.

## Results

### Type I error

The first step in comparing the eight methods for pathway analysis involved comparing the type I error rates of the eight methods on 500 sets that did not contain any truly causal SNPs (see Table 1). Although four of the eight methods controlled type I error well, the other four methods saw substantial type I error rates. We also created 500 additional null sets that did not contain any genes showing spurious association with the phenotype across the 200 replicates (see Methods section for details). All methods controlled type I error on these sets.

**Table 1:** Type I error rates across the five approaches for the 500 null sets and the 500 nonspurious gene sets

Pathway method	Across 500 null sets		Across 500 nonspurious gene sets	
	
	Nominal *α* = 0.05	Nominal *α* = 0.005	Nominal *α* = 0.05	Nominal *α* = 0.005
No gene-level aggregation				

WS	0.492	0.190	0.043	0.004
CMC	0.232	0.054	0.037	0.003

Gene-level aggregation				

WS-GSEA	0.048	0.004	0.001	0.000
WS-KS	0.040	0.003	0.000	0.000
WS-Fisher	0.429	0.244	0.010	0.004
CMC-GSEA	0.063	0.007	0.002	0.000
CMC-KS	0.044	0.004	0.004	0.000
CMC-Fisher	0.484	0.235	0.048	0.006

WS, weighted sum; CMC, combined multivariate collapsing; GSEA, gene set enrichment analysis; KS, Kolmogorov-Smirnov test; Fisher, Fisher’s combined probability test.

### Power

**Figure 1:**
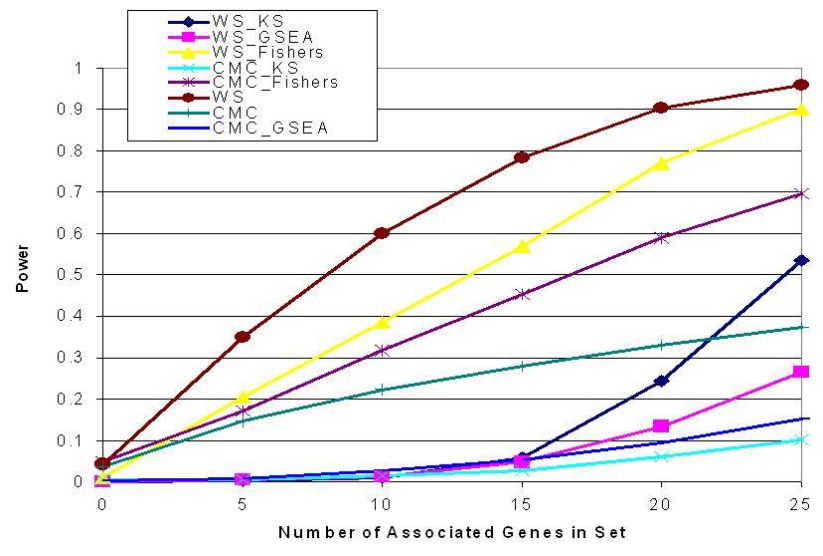
Power of pathway analysis methods across gene sets with varying numbers of associated genes

Next we explored the power of the eight different methods. Because of inflation of the type I error rate as a result of genes showing spurious association with the phenotype, we chose to analyze the power of only the 500 sets containing truly causal genes and noncausal genes that also did not show spurious association with the phenotype. Figure 1 illustrates the power of the eight methods across gene sets containing between 0 and 25 causal genes in the set of 25 genes. Direct application of the WS approach showed better average power than all other methods across sets containing from 5–25 causal genes. The WS-Fisher method and the CMC-Fisher method substantially outperformed the other methods. All methods showed power gains as the number of genes associated with the phenotype increased. Because the sets do not contain spuriously associated genes, all methods show accurate control or overly conservative control of the type I error rate.

### Relationships between power and set characteristics

For each set and method combination, we calculated the number of times out of 200 that the set was identified as significant (*α* = 0.05) for the method. For each set, we then looked at which method identified the set as significant the most times out of 200. The WS-GSEA, WS-KS, CMC-GSEA, and CMC-KS methods were not optimal for any of the 500 sets, and so we eliminated those methods from further analysis. Furthermore, the CMC method was best for only five sets, and the average increase in power for the remaining three methods was only 2.9% (SD = 1.8%). Because this was only a marginal increase, we eliminated the CMC method from further consideration, leaving three methods under consideration: WS, WS-Fisher, and CMC-Fisher, which were also the three methods that consistently yielded the highest average power across all sets (see Figure 1).

Each of the three methods had particular sets for which it was optimal: The WS method yielded the highest power for 429 of the 500 sets, the WS-Fisher method for 44 of the 500 sets, and the CMC-Fisher method for 27 of the 500 sets. For the 27 sets for which the CMC-Fisher method was best, the average power increase was 8.0% (SD = 7.9%) compared to the WS method and 13.4% (SD = 10.6%) compared to the WS-Fisher method. For the 44 sets for which the WS-Fisher method was best, the average power increase was 9.8% (SD = 9.7%) compared to the WS method. In the rest of this section, we attempt to characterize the sets found to be optimal by each method to provide insight into the strengths and weaknesses of each approach.

A logistic regression model was fitted to predict whether the WS method was the best. The model used five explanatory variables: (1) number of causal genes in the set, (2) number of causal SNPs in the set, (3) total number of SNPs in the set, (4) average MAF for associated genes in the set (MAFs for causal SNPs were first summed within genes), and (5) weighted risk score (sum of pairwise products of MAF and *β* for each causal SNP in the set; *β* is the true risk of the SNP on the phenotype). In the model, two of the five variables were significant (*p* < 0.01): the weighted risk score (*p* = 1.5 × 10^−7^) was negatively associated, whereas the number of causal SNPs (*p* = 4.8 × 10^−7^) was positively associated. Thus overall the WS method did better than the Fisher methods when the number of causal SNPs in the set was higher and the weighted risk score was lower, this being a situation of consistently small associations in the set. The Fisher methods did better in situations in which there were only a few strong associations in the set.

## Discussion

Overall, we find substantial differences in power and type I error rates between the different methods, with the WS, WS-Fisher, and CMC-Fisher methods having the highest power while controlling the type I error rate. However, type I error control occurs only with the elimination of spurious genes, which have been identified by others [11], and may be associated with the phenotype as a result of gametic phase disequilibrium and population stratification. Two popular methods of conducting gene set analysis (GSEA and the KS test) perform poorly relative to Fisher’s method and direct application of rare variant statistics to sets of SNPs.

As noted, when spuriously associated genes are removed from sets, type I error rates are well controlled by all methods (including the WS and WS-Fisher methods), suggesting that if spurious associations are better handled by rare variant methods, type I errors should be well controlled. As shown by Luedtke et al. [11], proper handling of population stratification eliminates some of the spurious associations. However, some spurious association still remains, and it tends to be associated with genes that show higher gametic phase disequilibrium with causal genes. Although some methods can handle population stratification, other methods are needed to control gametic phase disequilibrium. It is important to note that our results suggest a lack of control of the type I error rate in practice. Furthermore, we note that the apparent overconservative nature of some of the methods for sets that eliminate spurious genes is due to the elimination of genes, suggesting that spurious association reduces the amount of variability in noncausal genes.

## Conclusions

Although the WS method outperforms the WS-Fisher and CMC-Fisher methods in the aggregate, the Fisher methods improve their relative performance when the number of causal SNPs is low and the weighted risk score of the set is high. This situation occurs when a few strongly associated genes or SNPs are present in a set but most of the set is not associated with the phenotype. Further analysis with a more comprehensive set of simulated and real sets is needed to fully explore the advantages and disadvantages of the WS method relative to the WS-Fisher and CMC-Fisher methods. It is particularly important to note that the Fisher methods may still be necessary and useful for pathway analysis because in many real-life applications of pathway analysis only a small fraction of a set may actually be associated with the phenotype.

## Competing interests

The authors declare that there are no competing interests.

## Authors’ contributions

NT and AB designed the study and directed the research. AP, AS, AL and SP implemented the study and analyzed results. AP, AS and NT drafted the manuscript.
